# Editorial: COVID and Tropical Diseases – Intersection of Policy and Science

**DOI:** 10.3389/fitd.2022.861715

**Published:** 2022-05-25

**Authors:** Kathryn Shaw-Saliba, Herman Kosasih, Chuen-Yen Lau

**Affiliations:** 1 National Institute of Allergy and Infectious Diseases, National Institutes of Health, Bethesda, MD, United States; 2 Indonesia Research Partnership on Infectious Disease (INA-RESPOND), Jakarta, Indonesia; 3 HIV Dynamics and Replication Program, Center for Cancer Research, National Cancer Institute, National Institutes of Health, Bethesda, MD, United States

**Keywords:** COVID – 19, tropical disease, public policy, Science, diagnostics, religion

COVID-19 poses unique threats to tropical disease endemic regions, many of which are resource limited. Local experience with emerging and re-emerging infections may inform strategies to mitigate the impact of COVID-19, which can interact with tropical diseases to affect transmission, severity, and long-term impacts of both diseases. Articles in this Research Topic will inform infectious disease public health management in tropical regions.

Ability to diagnose SARS-CoV-2 rapidly and accurately is essential to controlling spread of COVID-19. Diagnosis is challenging when healthcare infrastructure is inadequate. Diarra et al. describe the coordinated utilization of four research laboratories for SARS-CoV-2 diagnostics in Mali. Pre-existing institutional research capacity resulting from decades of capacity building and collaborative experience from the 2014–2015 Ebola outbreak, allowed Mali to develop a coordinated approach prior to the introduction of SARS-CoV-2. Challenges included the need to import testing supplies and dependence upon donations, threatening long-term sustainability. The research centers are located in Bamako and rely largely on molecular diagnostics, limiting ability to rapidly diagnose cases from outlying communities. Despite these challenges, the institutions continue to test specimens daily from across Mali two years into the pandemic. In light of the diagnostics program success, one of the institutions was also recruited to support the Malian national COVID-19 vaccination program. The Mali experience demonstrates how research infrastructure can be mobilized for response in resource limited settings (1–7).

In addition to the challenges of diagnosing acute infections, studies using serological (antibody) assays designed to determine what proportion of the population has been infected and evaluate vaccine immunogenicity have encountered unique complexity in tropical disease endemic regions. As evidenced in Woodford et al. in Mali and referenced in Yek et al. in Southeast Asia, previous or current infection with malaria can result in false positive cross-reactivity (8–15). This may apply to other common tropical diseases and polyclonal B cell activation. This cross-reactivity has not been demonstrated to translate to functionality as evidenced by negative findings on neutralizing antibody assays. Therefore, false positivity can impact interpretation of immunogenicity and policy decisions as described in Yek et al. Woodford et al. describe important methods for dealing with false positive cross-reactivity including setting regional standards based on pre-pandemic specimens.

Yek et al. also address disease management when COVID-19 intersects with other endemic and epidemic tropical diseases in Southeast Asia. Occurrence of the COVID-19 pandemic on the background of contemporaneous circulation of endemic infection, including malaria and dengue, in Southeast Asia produced numerous challenges. Cross reactivity of COVID-19 and malaria serodiagnostics (and potential cross reactivity associated with other common viruses including dengue and other coronaviruses), associated over-estimation of pre-existing immunity, and slow vaccine roll-out may have influenced the epidemiology of COVID-19 in Southeast Asian countries. Meanwhile, COVID-19 public policies may have increased exposure to other infections in rural areas when lockdown of urban employment sites caused migration out of cities. Furthermore, unevidenced repurposing of antiparasitic and antiviral drugs may have detracted from resources for ongoing health programs and facilitated selection of drug-resistant organisms. The authors demonstrate the need for a coordinated healthcare infrastructure and suggest some essential components in their conclusion.

Intersection of the COVID-19 pandemic with other endemic diseases is also evidenced in Hariadi et al. and Krismawati et al.. Hariadi et al. describe a case of COVID-19 and dengue co-infection that occurred in Indonesia early in the pandemic. The patient was a presumed dengue case, who was tested for COVID-19 after meeting screening criteria. Relatively rapid identification of COVID-19 facilitated enactment of appropriate precautions. This case highlights the need to prioritize accessibility of rapid diagnostics in resource limited settings, and the need to test for both the pandemic disease and known endemic diseases.

In Krismawati et al., the impact of the COVID-19 pandemic on control of other endemic neglected tropical diseases is further examined. In Papua, Indonesia, new diagnoses of leprosy decreased during 2020 while severity of new cases increased, indicating that the COVID-19 pandemic negatively impacted ability to detect and treat leprosy. This is broadly applicable to other infectious diseases and regions and may reflect factors such as inability or hesitancy to seek care and shunting of health and diagnostic resources to COVID-19 (16, 17). As a parallel example, HIV testing rates, antiretroviral treatment access, clinical visits and screening for other sexually transmitted infections decreased during the COVID-19 pandemic. Approaches to HIV care have been adjusting to include more telehealth encounters and alternative strategies to ensure adequate access to medication. It has also been suggested that combining COVID-19 and HIV testing could improve rates of HIV detection (18).

While Yek et al. and Krismawati et al. raise important points regarding COVID-19 treatment, prevention, and acceptance in diverse tropical populations, Mardian et al. focus on factors affecting COVID-19 vaccine acceptance according to *Sharia* (Islamic Law). Religious restrictions have long played a role in healthcare acceptance – Jehovah’s witnesses do not accept blood transfusions and Christian scientist rely heavily on prayer for healing. *Sharia* forbids Muslims from consuming certain products, particularly those derived from swine. Mardian et al. demonstrate that COVID-19 vaccination is consistent with the importance placed on safety of human life in Islamic jurisprudence. They also explain how exceptions to consumption requirements can be enacted by *Fatwa* councils. Concepts in this paper go a long way in supporting COVID-19 vaccine acceptability.

The collection of papers in this Research Topic provides insight on the challenges associated with prevention, detection, and management of COVID-19 in tropical settings and beyond. The importance of rapid diagnostics for management of the pandemic is highlighted from the individual to regional level. Need to consider the reciprocal impact of co-circulating tropical diseases on diagnosis and management in public health responses is also demonstrated. Conversely, resource diversion to COVID-19 can impair other disease management programs. International partnerships can provide support in urgent situations, but sustainable strategies must be nurtured. Lastly, the influence of religious, social, and personal perspectives on community acceptance of COVID-19 public health strategies cannot be overlooked. Figure 1 shows policy components that can be used to address COVID-19 and other pandemics in tropical disease endemic regions.

## Figures and Tables

**FIGURE 1 |:**
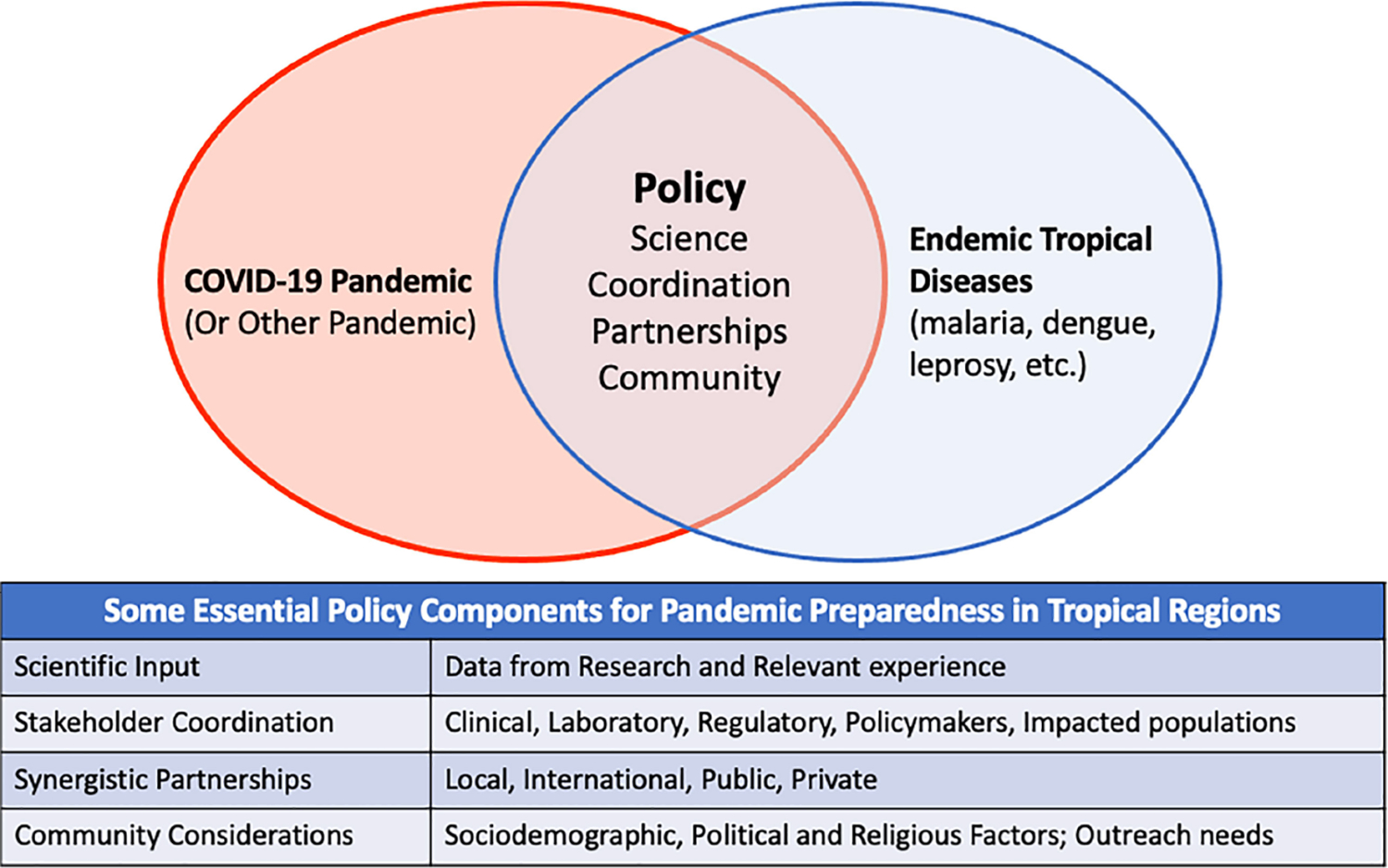
Conceptual Diagram showing policy components that can facilitate addressing COVID-19 and other pandemics in regions endemic for tropical infections. The table shows details of the specific policy components.
